# How foot-and-mouth disease virus receptor mediates foot-and-mouth disease virus infection

**DOI:** 10.1186/s12985-015-0246-z

**Published:** 2015-02-03

**Authors:** Guangxiang Wang, Yanhua Wang, Youjun Shang, Zhidong Zhang, Xiangtao Liu

**Affiliations:** State Key Laboratory of Veterinary Etiological Biology, Lanzhou, 730046 China; National Foot-and-Mouth Disease Reference Laboratory, Lanzhou, 730046 China; Key Laboratory of Animal Virology of Ministry of Agriculture, Lanzhou, 730046 China; Lanzhou Veterinary Research Institute, Chinese Academy of Agricultural Sciences, Lanzhou, 730046 China

**Keywords:** Foot-and-mouth disease virus, Receptor, Infection

## Abstract

This study reviews the FMDV receptor-binding domain, integrin receptors, and heparan sulfate receptors to provide references for studies regarding the mechanisms underlying FMDV infection.

## Background

Foot-and-mouth disease (FMD) is a highly contagious and fulminating infectious disease in mammals. Although its mortality in adult animals is not high, its mortality in young animals is relatively high. After infection, the morbidity in animals reaches almost 100%. FMD is listed as the number one infectious disease by the Office International des Epizooties (OIE) in France [1]. Foot-and-mouth disease virus (FMDV) belongs to the family *Picornaviridae*, which has 7 serotypes (O, A, C, SAT1, SAT2, SAT3, and Asia I) and many subtypes [2]. FMDV infects many cloven-hoofed animals and causes serious economic, political, and social problems [3]. Currently, no effective drug for treating FMD; thus, its danger is obvious [4,5].

Host cell adsorption is a prerequisite for FMDV to infect cells. This adsorption process depends on host cell receptors. Viruses initiate the infection process through binding to specific receptors on the cell surface of susceptible host cells. Host tissues and specific cell surface receptors determine the invasion routes and dissemination methods of viruses as well as the pathogenic features of hosts [6]. Studies regarding the FMDV ligand and cell receptors help to not only understand FMDV cell receptors but also to elucidate the infection routes, replication process, pathogenesis, and host tropism of FMDV, providing scientific bases for the prevention, control, and treatment of FMD. This article reviews the progress of studies regarding the FMDV receptor-binding domain (ligand) and its receptors to provide references for studying mechanisms underlying FMDV infection and for the prevention and treatment of this disease.

## Review

### FMDV receptor-binding domain (ligand) and FMDV infection

The genome of FMDV is a single-stranded, positive-sense RNA, which serves as both mRNA and the template for negative-stranded RNA. The genome of FMDV is composed of approximately 8,500 nucleotides (nt) and has the following 3 components: a 5′-untranslated region (5′-UTR), an open reading fragment (ORF), and a 3′-UTR. The ORF encodes a polyprotein that forms the following 4 fragments after the primary cleavage: the L fragment, the P1/2A fragment, the P2BC fragment, and the P3 fragment. After the secondary cleavage and the maturation cleavage, 4 structural proteins (VP4, VP2, VP3, and VP1) and 8–9 non-structural proteins (Lab/Lb, 2A, 2B, 2C, 3A, 3B1, 3B2, 3B3, 3C, and 3D) are formed [2].

The capsid proteins of FMDV are composed of 60 copies of the 4 structural proteins VP1, VP2, VP3, and VP4 (also known as 1D, 1B, 1C, and 1A) of FMDV and display an icosahedral structure [7]. One copy of VP1, VP2, VP3, and VP4 proteins forms a protomer; 5 protomers assemble into a pentamer; and 12 pentamers form a complete viral capsid [8,9]. The capsids of FMDV particles have many small holes that are surrounded by 5 identical protomers composed of VP1, VP2, VP3, and VP4. VP1 is close to the small hole, VP2 and VP4 are at the distal end, and VP4 is completely inside of the capsid. The most obvious feature of the surface of FMDV particles is a surface-protruding G-H loop formed by the βG and βH chains of residues 140–160 of VP1. The G-H loop contains a highly conserved arginine-glycine-aspartate (Arg-Gly-Asp, RGD) sequence. This sequence is an important neutralizing site of FMDV [10]; this sequence is also the primary component of the cell adsorption site and can interact with integrin receptors on the cell membrane to mediate the initiation of virus infection [7,11]. Except for VP4, the capsid proteins VP1, VP2, and VP3 are all associated with antigenicity and can interact with the RGD-directed integrin protein subunits and with heparan sulfate proteoglycans (HSPGs) on the cell surface. The variations of the amino acid residues in FMDV capsid proteins that form viral particles not only can affect the adsorption ability between viruses and cell receptors but also may affect the biological characteristics of viruses [10,12,13].

The RGD sequence of the G-H loop in the VP1 capsid protein of FMDV has been shown to form an extremely stable complex with cellular integrin proteins, and this complex is resistant to EDTA. The replacement of specific amino acids demonstrated that the stability of binding to αvβ6 depends on a helical structure close to the RGD sequence. The leucine residues at RGD + 1 and RGD + 4 sites are the key positions for this stable interaction. The stability of this complex helps to increase the possibility of virus adsorption and internalization, thus increasing FMDV infectivity [14]. The study by Escarmis et al. [15] regarding the crystallographic structure of viral particles using X-ray scattering demonstrated that the G-H loop of VP1 was highly disordered. When the viral capsid interacted with the antigen-binding fragment (Fab), this loop protruded from the viral capsid. Then, the RGD sequence in the G-H loop interacted with the cellular integrin receptors, and the virus infected cells through internalization.

In addition to the RGD sequence, FMDV can also utilize other tripeptide sequences similar to the RGD sequence to interact with receptors. Martinez et al. [16] demonstrated that when the RGD sequences were changed to REG sequences, FMDV could still replicate normally in its susceptible cells. Therefore, these authors speculated that FMDV might use other unknown mechanisms to recognize cells. In addition, FMDV is an RNA virus, which has the characteristics of quasispecies in nature. After being passaged for several generations in chicken embryos or cells or under external conditions, such as vaccine pressure, FMDV can produce many different antigenic variants. These variant strains can cause infection independent of the RGD sequence. For example, when the FMDV strain C-S8c1 was passaged in cells for 100 generations, a mutant strain containing the Arg-Gly-Gly (RGG) receptor-binding sequence appeared. When this RGG containing mutant strain was passaged for 50 generations in cells, a mutant strain containing the Gly-Gly-Gly (GGG) receptor-binding sequence appeared. These results indicated that FMDV could produce viable and infectious virions that did not depend on the RGD sequence in addition to primarily depending on the RGD sequence during the process of evolution. Furthermore, these viral variants could use a currently unknown method to interact with cells, causing adsorption and infection [17-19]. However, studies regarding the function of these mutated receptor binding sites and cell entry, followed by the recognition of cellular protein molecules, remain lacking; therefore, whether variations in these receptor-binding sites cause changes in host tropism remains unclear [20].

### Integrin-mediated FMDV infection

#### The mechanism underlying integrin-mediated FMDV infection and integrin proteins that mediate FMBV infection

Integrins are a family of extensively distributed cell surface receptors. Members of this family are all heterodimeric transmembrane glycoproteins containing α and β subunits formed by non-covalent interactions. The subunits of integrins belong to the type I transmembrane proteins, which include a large extracellular domain, a small transmembrane domain, and a cytoplasmic domain. Each chain consists of an extracellular region, a transmembrane region, and a cytoplasmic region. The spherical region formed by the N-terminal α and β chains is the extracellular ligand-binding domain. Integrins primarily mediate cell adhesion and signal transduction and participate in many physiological functions, such as cell growth, development, differentiation, and apoptosis [21]. Some studies have demonstrated that integrin-mediated FMDV infection occurs through clathrin-dependent endocytosis; acidified endocytic vesicles cause rapid cleavage of the viral capsid protein structure, thus causing RNA release through a currently unknown mechanism [22]. At least 18 known different α and β subunits that form 24 different αβ heterodimers have been identified [23]. Only 8 integrins (αvβ1, α5β1, αvβ3, αvβ8, αvβ5, αvβ6, α8β1, and αII bβ3) can interact with the RGD sequence in the G-H loop of FMDV VP1 [24]. At least 4 integrins (αvβ1, αvβ3, αvβ6, and αvβ8) can be used as FMDV receptors to mediate FMDV infection [25-28].

#### αvβ3 integrin-mediated FMDV infection

**Figure 1:**
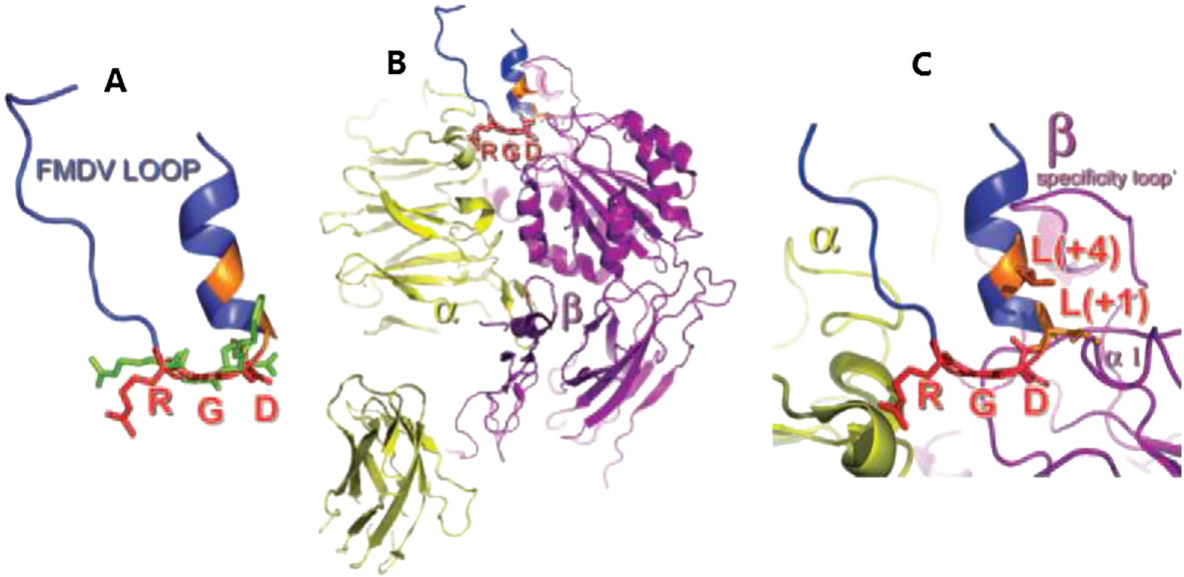
**Integrin binding domain with the G-H loop and RGD sequence of VP1 [** 29 **].** Panel **A** shows the FMDV VP1 GH loop. Panel **B** shows the FMDV VP1 GH loop (as in panel **A**) in the context of αvβ3. The loop is predicted to bind in the crevice between the αv β-propeller domain (yellow/green ribbon) and the ligand-binding domain of β3 (purple/pink ribbon). Panel **C** shows a close up of panel B with the side chains of Leu (RGD + 1 and RGD + 4) shown as orange sticks projecting from the same face of the 3_10_ helix.

αvβ3 is the first discovered FMDV receptor [25]. In 1995, Berinstein et al. [25] found that the anti-serum against αvβ3 of the human vitronectin receptor and a monoclonal antibody against αvβ3 could both block FMDV infection in susceptible cells; therefore, αvβ3 was confirmed to be a cellular receptor for this virus. X-ray scattering studies have demonstrated that the extracellular portion of αvβ3 integrin has 12 different structural domains, 4 of which are in the α subunit and 8 of which are in the β subunit. These domains form a functional molecule with an oval head and two tails [29] (Figure 1). The tails are the active sites and are associated with the cellular regulatory mechanism. The BA domain contains a metal ion-dependent adhesion site close to a calcium-binding site, which has regulatory functions [30]. In 2001, Neff et al. [31] demonstrated that the truncation or extension of the cytoplasmic region of αv and β3 subunits, including the deletion of the entire cytoplasmic region, had no or minor effects on αvβ3 as a receptor for FMDV. In addition, FMDV replication was inhibited by an αvβ3 antibody but not by antibodies of other RGD-directed integrins including αvβ5, αvβ1, and αvβ6. αvβ6 antibodies inhibit in one case [27] but not the other. In 2005, Monaghan et al. [32] monitored αvβ3 and αvβ6 expression in bovine epithelial cells of FMDV target tissues using immunofluorescence confocal microscopy and real-time RT-PCR. The results indicated that the surface of epithelial cells with a high FMDV replication level during natural infection expressed αvβ6 but not αvβ3 and that FMDV infected cells in interdigital skin also expressed αvβ6. These data indicated that αvβ3 was not a major receptor of tissue tropism for FMDV to infect target epithelial cells. The αvβ3 integrin protein might be an adsorption receptor for viruses because the deletion of the cytoplasmic domain of the αv or β3 subunit did not affect the efficiency of viral infection; thus, other cell surface molecules might serve as co-receptors and play a role in the internalization process of viruses [31].

#### αvβ6 integrin-mediated FMDV infection

In 2000, Jackson et al. [26] demonstrated that human colon carcinoma SW480 cells nonpermissive for FMDV under normal conditions became susceptible to FMDV after transfection with human β6 integrin cDNA. This infection could be suppressed by an anti-αvβ6 monoclonal antibody, and this interaction was dependent on the RGD sequence. Therefore, αvβ6 was identified as another functional receptor for FMDV. The β6 subunit can only form a heterodimer, αvβ6; αvβ6 belongs to a subgroup of the integrin family. This subgroup can use a common RGD sequence [32]. αvβ6 is only expressed in epithelial cells; however, its expression levels in different epithelial cells vary. αvβ6 has high expression levels in the epithelial cells of the macula densa and endometria as well as in tongue epithelial cells and salivary glands; however, αvβ6 has low expression levels in skin and lung epithelial cells [33,34]. Brown et al. [35] cloned the sheep β6 subunit and confirmed that αvβ6 expression was low in sheep epithelial cells using RT-PCR and immunohistochemistry. αvβ6 could be detected in the epithelial cells of the airways, oral cavity, gastrointestinal tract, kidney, sweat glands, and hair follicular sheath and in the epidermis of the pedal coronary band but not of normal skin. In addition, αvβ6 was highly expressed in ovine tonsillar crypt epithelial cells and in bovine and ovine tracheal epithelial cells, indicating that tracheal epithelial cells might be the portal of entry in ruminants at the early stage of FMDV infection. The results of Du et al. [36] were similar to those results of Monaghan et al. [37] demonstrating that αvβ6 is primarily distributed in the spinous layer of epithelial cells and that αvβ3 is only expressed at low levels in the vascular system. These results suggested that αvβ6 might be the major functional receptor determining FMDV tissue tropism. The tissue distribution of αvβ3 seemed to be unrelated to the tissue tropism of viruses; however, its function at the late stage of viral infection could not be excluded.

αvβ6 is a receptor that interacts with FMDV; however, the process of its involvement in the infection of cells by viruses remains unclear. In 2001, Miller et al. [38] partially or completely deleted the cytoplasmic domain of β6 and found that its binding to viruses was not affected but that viral infection was affected, indicating that this region plays an important role after virus interaction. In 2005, Berryman et al. [39] and O′Donnell [22] both simultaneously reported that FMDV infection of αvβ6-expressing cells was associated with the endocytosis function of integrins in the porous structure and that this endocytosis function was mediated by clathrin. Together with the acidification of endosomes, this endocytosis function induced the degradation of the viral capsid structure to release genomes. This mechanism remains unclear, and no lipid raft-dependent caveolin or other endocytosis pathway appeared to be involved. Some studies have demonstrated that αvβ6 integrin not only functions in the cell adsorption process of FMDV but also plays important roles in the process of virus uncoating and replication [39]. Neff et al. [31] deleted most amino acid residues in the C-terminus of the cytoplasmic domain of human β6 or in the core region containing the “NPLY” sequence and demonstrated that the mutant β6 could not normally mediate FMDV infection. These results confirmed the important role of the conserved “NPLY” sequence of the integrin cytoplasmic domain in cell signal transduction. In addition, Miller et al. [38] demonstrated that the β6 subunit could facilitate cell growth because of the C-terminal 11 amino acids. In comparison, soluble αvβ3 could not bind to particular type A or type O viruses; however, these two types of viruses could bind to particular integrins, indicating that the binding between FMDV and αvβ3 was a low affinity interaction. In addition, soluble αvβ3 could not inhibit virus infection. Incubation with soluble αvβ6 could significantly inhibit the interaction between the A12 or O1 type FMDV and BHK cells, whereas incubation with soluble αvβ3 only had low levels of inhibition. These results indicated that at least *in vitro* soluble receptors could interfere with FMDV infection of cells, establishing foundations for the utilization of soluble receptors and for the development of anti-integrin protein drugs for the control of FMD [40].

#### αvβ1 integrin-mediated FMDV infection

In 2002, Jackson et al. [27] discovered that FMDV could not infect CHOB2 cells with αv subunit expression deficiency under normal conditions. After transfecting human αv cDNA into CHOB2 cells, these cells began to express heterologous αvβ1 integrin (human αv/hamster β1) and became susceptible to FMDV. These results confirmed that αvβ1 can serve as a functional receptor for FMDV. αvβ1-mediated virus adsorption could be blocked by monoclonal antibodies against αv or an RGD sequence containing a short peptide, which confirmed that the αv subunit contained the major virus-binding domain [27]. The expression of αvβ1 integrin is restricted in some specific cells; for example, malignant tumor cells, smooth muscles, and the central nervous system express αvβ1 [41]. Some cells express many αv and β1 subunits but do not express αvβ1 heterodimers, causing some difficulties in αvβ1 research.

αvβ1 integrin is ineffective under physiological concentrations of calcium and magnesium ions. However, treating cells with manganese ions or with an anti-β1 antibody with an activation function significantly increased the infectivity of FMDV in cells. Therefore, the ability of FMDV to utilize αvβ1 as a virus receptor might depend on a cellular regulatory mechanism underlying the interaction between integrin protein molecules and ligands [27,39].

#### αvβ8 integrin-mediated FMDV infection

In 2004, Jackson et al. [28] transfected human β8 cDNA into SW480 cells nonpermissive for FMDV infection. This cell line expressed αvβ8 on the cell surface and became susceptible for FMDV. Treating these cells with specific monoclonal antibodies against αvβ8 heterodimers or αv subunits could block FMDV infection. Therefore, αvβ8 was confirmed as the fourth discovered functional integrin receptor for FMDV, after αvβ3, αvβ6, and αvβ1. In addition, Cambier et al. [42] in 2000 and Fjellbirkeland et al. [43] in 2003 both identified αvβ8 expression in airway epithelial basal cells in mammals, which is the primary proliferation site of FMDV. These results indicated that αvβ8 might function at the early stage of FMDV infection and might affect the tropism and pathogenesis of viruses.

To study the roles of different integrins in the viral infection process, Duque and Baxt [44] cloned bovine αv, β1, β3, β5, and β6 integrin subunits and transiently transfected these subunits into cells to compare the utilization rates of αvβ1, αvβ3, αvβ5, and αvβ6 by three strains of serotype A and by two strains of serotype O FMDV. The results demonstrated that the three strains of type A viruses could utilize αvβ3 and αvβ6 with relatively high efficiency and could utilize αvβ1 with moderate efficiency, whereas the two strains of type O viruses utilized αvβ6 and αvβ1 with higher efficiency than αvβ3. All viruses could only replicate at low levels in cells expressing αvβ5. Experiments in which the ligand-binding domains among the β subunits were exchanged indicated that this ligand-binding region was helpful for elucidating the differences in integrin utilization by different strains of viruses. Infection with different strains of viruses might result in different severities of diseases; however, in most cases, the clinical symptoms of the same species did not have significant difference. This observation might be associated with the expression patterns and expression levels of FMDV integrin receptors among different species.

### Heparan sulfate-mediated FMDV infection

Heparan sulfate (HS) is a highly sulfated glycosaminoglycan (GAG). GAGs, which are long unbranched polysaccharides consisting of a repeating disaccharide unit (hexosamine, hexuronic acid, or galactose), extensively exist in the cell membrane and in the extracellular matrix. Under physiological conditions, the N-sulfate group or the O-sulfate group in the HS carbon chain provides many negative charges to the sugar chain. This sulfated polysaccharide sequence structure provides HSPGs with not only their anionic feature and high density negative charges but also the ability to interact with other extracellular substances, including viruses [45]. In 1996, Jackson et al. [46] demonstrated that heparin could specifically block FMDV infection in cultured cells and that heparin-treated cells had significantly reduced plaque formation after FMDV infection. In addition, FMDV could not infect HS-deficient cells. These results confirmed that HS on the cell membrane is a receptor for FMDV.

**Figure 2:**
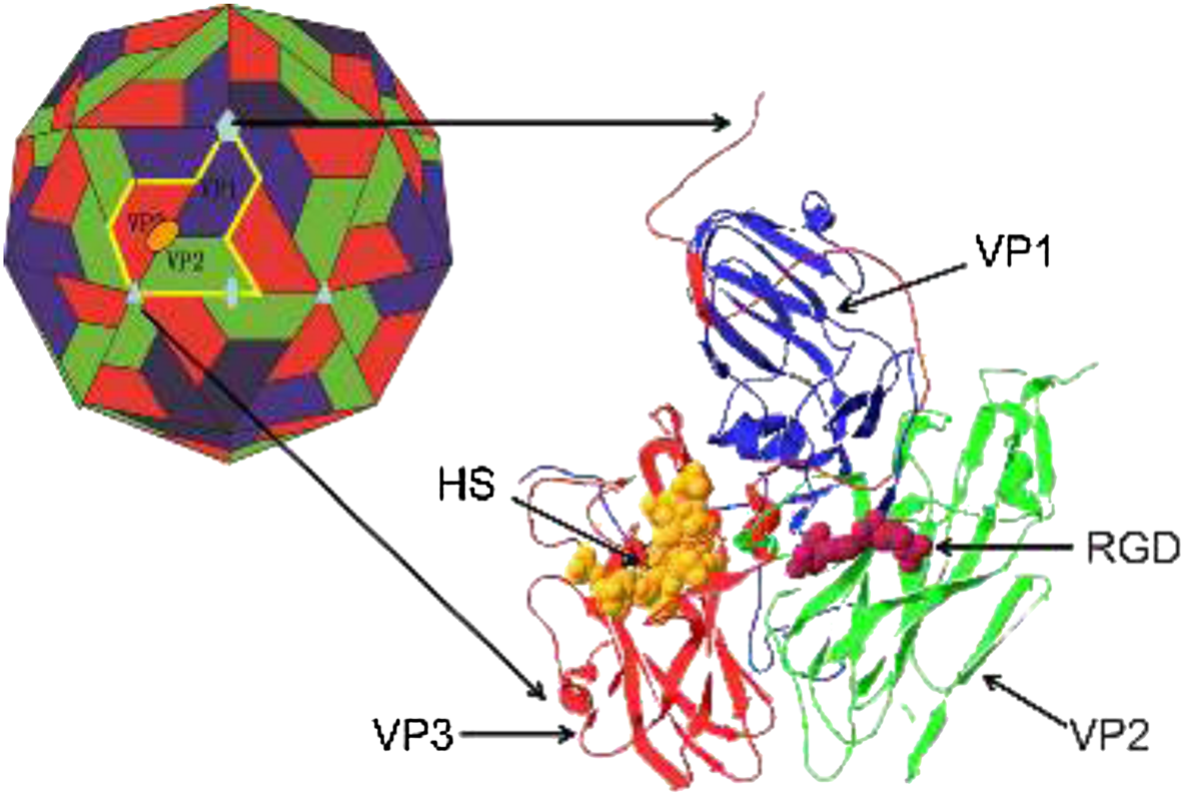
**Model of the interaction of FMDV with heparan sulfate receptor [** 7 **,** 49 **,** 50 **].** The small orange oval indicates the HS-binding site. The RGD integrin-binding motif is shown in a magenta surface representation [50].

HS was originally considered a co-receptor for the O type FMDV strain to enter cells [46]. Subsequent studies have demonstrated that other serotypes (such as A, C, Asia1, and SAT1) of FMDV could also bind to HS [47]. Baxt [48] found that the A type FMDV could not bind to CHO cells only expressing the HS receptor, whereas the O type FMDV VP3 (Arg56) mutant strain could replicate in CHO cells. In contrast, the VP3 (His56) mutant strain could not replicate. These results indicated that HS could have a direct electrostatic adsorption function with the positively charged arginine residue at position 56 in the VP3 protein. Other studies have demonstrated that the C-terminal amino acids at positions 201–211 of VP1 might participate in the adsorption process between viruses and cells. The sequences of the C-terminal 201–211 region of VP1 are similar to the heparin-binding site of vitronectin, suggesting that this region might interact with HS. However, the crystal structure of the FMDV and HS complex did not display the same result. The histidine at position 195 and the lysine at position 193 of VP1 in the O1BFS and A10 strains were both linked with HS, suggesting that the C-terminus of VP1 might facilitate the interaction between these residues and HS. The analysis of the crystal structure indicated that the HS-binding site of FMDV was approximately 15 Å from the RGD sequence; the spatial locations of these two were extremely close [7,49,50] (Figure 2), suggesting that integrin and HS receptors might simultaneously interact with FMDV to mediate FMDV infection [51]. Whether this situation can be applied for all FMDV still awaits further validation. Jackson et al. [46] once considered that HS was the first step for virus-cell interaction, followed by virus-integrin interaction. Some scholars have demonstrated that the HS receptor mediated FMDV infection through Caveola-dependent endocytosis pathway [52,53]. However, because different strains of FMDV can use different types of receptors, evidence demonstrating the functional relation between HS and integrin remains lacking. Many studies have indicated that HS might be a replacement receptor for FMDV or an alternative pathway for entry into cells after virus infection [54].

### The third group receptor-mediated FMDV infection

In addition to utilizing integrins and HS for FMDV receptors, a hypothesis regarding a third group of receptors for FMDV was proposed. For example, Baxt et al. [55] found that FMDV could infect macrophages through Fc receptor-mediated adsorption. Rieder et al. [56] utilized genetic engineering to fuse a single-chain antibody (scab) that could interact with viruses to intercellular adhesion molecule 1 (ICAM 1) to produce a new FMDV receptor (scAb/ICAM 1). FMDV A12 could infect CHO cells expressing this replacement receptor but not normal CHO cells. After the RGD sequence in the FMDV C-S8c1 strain was mutated into RGG, this strain could obtain viability in K653 cells that did not express αvβ3. Zhao et al. [57] constructed a chimeric virus using cDNA from the FMDV A12 infectious clone O/CHA/90 and its cell-adapted strain vac-O/CHA/90. When the RGD sequence was artificially mutated into KGE, the chimeric virus could still grow in cultured cells after HS utilization was excluded. Other discovered natural mutations in the cell adsorption site of FMDV included GGD, TGD, RDD, PGD, KGN, RSG, KGD, and IGD [20,47,54]. These experimental results indicated that a third group of FMDV receptors might mediate FMDV infection.

## Conclusions

Receptors are the major determinant factors for the tropism and pathogenesis of viruses. Viruses may utilize different receptors at the different stage of viral pathogenesis. FMDV is the only virus in the *Picornaviridae* family that can utilize 4 different integrin proteins, αvβ1, αvβ3, αvβ6, and αvβ8, to mediate infection. However, the function of each receptor in the process of host cell infection by FMDV remains unknown. In addition, under the conditions of adapted cell culture, FMDV obtains the ability to utilize HS as its receptor. Although this feature is associated with reduced virulence, yet unidentified receptor may be associated with the infection of cells by FMDV. In addition, different serotypes of FMDV strains have different efficiencies for receptors during the process of infecting cells. Therefore, investigating FMDV receptors provides experimental data for understanding the mechanism underlying the infection of cells by different serotypes of FMDV, the influencing factors (including the expression profiles of host cell receptors and virulence-related genes, the structural changes of the receptor-recognition regions, and the viral load), and the evaluation of the virulence of FMDV epidemic strains and their host ranges. These investigations also establish a theoretical foundation for elucidating the molecular mechanisms underlying host tropism and cross-species FMDV infection. Investigating the mechanism underlying the utilization of receptors to mediate cell infection by FMDV can theoretically unravel the structure of FMDV receptors and their roles in the process of FMDV infection of host cells, thus facilitating the design of corresponding chemical drugs to block the interaction between FMDV and host cells and providing new insights and methods for the prevention and control of FMD.
